# Circulating tumor DNA predicts recurrence and survival in patients with resectable gastric and gastroesophageal junction cancer

**DOI:** 10.1007/s10120-024-01556-9

**Published:** 2024-10-05

**Authors:** Cecilie Riis Iden, Salah Mohammad Mustafa, Nadia Øgaard, Tenna Henriksen, Sarah Østrup Jensen, Lise Barlebo Ahlborn, Kristian Egebjerg, Lene Baeksgaard, Rajendra Singh Garbyal, Mette Kjølhede Nedergaard, Michael Patrick Achiam, Claus Lindbjerg Andersen, Morten Mau-Sørensen

**Affiliations:** 1 Department of Oncology, Copenhagen University Hospital, Rigshospitalet, Blegdamsvej 9, 2100 Copenhagen, Denmark; 2 Department of Molecular Medicine, Aarhus University Hospital, Palle Juul-Jensens, Boulevard 99, 8200 Aarhus N, Denmark; 3 Institute of Clinical Medicine, Faculty of Health, Aarhus University, Palle Juul-Jensens Boulevard 82, 8200 Aarhus N, Denmark; 4 Department of Genomic Medicine, Copenhagen University Hospital, Rigshospitalet, Blegdamsvej 9, 2100 Copenhagen, Denmark; 5 Department of Pathology, Copenhagen University Hospital, Rigshospitalet, Blegdamsvej 9, 2100 Copenhagen, Denmark; 6 Department of Surgery & Transplantation, Copenhagen University Hospital, Rigshospitalet, Blegdamsvej 9, 2100 Copenhagen, Denmark

**Keywords:** Circulating tumor DNA, Gastroesophageal cancer, Curative treatment, DNA methylation, Tumor biomarkers

## Abstract

**Background:**

Gastric and gastroesophageal junction (GEJ) cancer represents a significant global health challenge, with high recurrence rates and poor survival outcomes. This study investigates circulating tumor DNA (ctDNA) as a biomarker for assessing recurrence risk in patients with resectable gastric and GEJ adenocarcinomas (AC).

**Methods:**

Patients with resectable gastric and GEJ AC, undergoing perioperative chemotherapy and surgery, were prospectively enrolled. Serial plasma samples were collected at baseline, after one cycle of chemotherapy, after preoperative chemotherapy, and after surgery. ctDNA was assessed by a ddPCR test (TriMeth), which targets the gastrointestinal cancer-specific methylation patterns of the genes *C9orf50*, *KCNQ5*, and *CLIP4*.

**Results:**

ctDNA analysis was performed on 229 plasma samples from 86 patients. At baseline, ctDNA was detected in 56% of patients, which decreased to 37% following one cycle of chemotherapy, 25% after preoperative chemotherapy and 15% after surgical resection. The presence of ctDNA after one cycle of chemotherapy was associated with reduced recurrence-free survival (RFS) (HR = 2.54, 95% confidence interval (CI) 1.33–4.85, *p* = 0.005) and overall survival (OS) (HR = 2.23, 95% CI 1.07–4.62, *p* = 0.032). Similarly, ctDNA after surgery was associated with significantly shorter RFS (HR = 6.22, 95% CI 2.39–16.2, *p* < 0.001) and OS (HR = 6.37, 95% CI 2.10–19.3, *p* = 0.001). Multivariable regression analysis confirmed ctDNA after surgery as an independent prognostic factor (*p* < 0.001).

**Conclusion:**

ctDNA analysis has the potential to identify patients at elevated risk of recurrence, thus providing personalized treatment strategies for patients with resectable gastric and GEJ cancer. Further validation in larger cohorts and ctDNA-guided interventions are needed for future clinical use.

**Supplementary Information:**

The online version contains supplementary material available at 10.1007/s10120-024-01556-9.

## Introduction

Gastric and gastroesophageal junction (GEJ) cancer remains a significant global health burden, with more than 1.7 million new cases diagnosed annually, resulting in approximately 1.3 million deaths in 2020 [1, 2]. The predominant histological subtype is adenocarcinoma (AC), constituting approximately 90% of gastric and GEJ cancers [3]. Patients with localized gastric and GEJ AC are offered curatively intended treatment, including surgical resection and perioperative chemotherapy or preoperative chemoradiotherapy [4, 5]. Nevertheless, despite the curative intent, recurrence rates in patients with resectable gastric and GEJ AC remain high, resulting in a poor 5-year survival rate of less than 50% [6]. The core challenge lies in identifying patients at an elevated risk of disease recurrence due to minimal residual disease following curatively intended treatment. Current surveillance programs lack the sensitivity to identify patients at high risk of recurrence, thereby hindering the timely introduction of therapeutic interventions [7, 8]. Consequently, there is a critical need for sensitive biomarkers for early recurrence detection and assessment of prognosis.

Circulating tumor DNA (ctDNA) has emerged as a promising biomarker for cancer detection and monitoring of tumor burden in cancer patients [9–11]. ctDNA is released into peripheral blood and other body fluids through apoptosis and necrosis of tumor cells, representing a small fraction of the cell-free DNA (cfDNA) in patients with cancer. In recent studies of patients with bladder [12] lung [13], and colorectal cancers (CRC) [14], it has been shown that the presence of ctDNA postoperatively is associated with an increased risk of recurrence. In gastroesophageal cancer, the presence of ctDNA following resection has been proven to correlate with a poor prognosis and a higher rate of recurrence [15, 16].

Cancer-specific DNA methylation alterations hold great potential as ctDNA markers [17, 18]. In contrast to tumor-informed strategies, ctDNA methylation detection has the major advantage of being tumor-agnostic, thus eliminating the need for prior tumor tissue analysis. In our previous studies, we have demonstrated a high sensitivity of ctDNA detection using the TriMeth test, a tumor-agnostic multiplex droplet digital PCR assay targeting the gastrointestinal cancer-specific DNA methylation markers, *C9orf50*, *KCNQ5*, and CLIP4 [19, 20]. This approach proved effective for early detection of CRC as well as the recurrence of CRC [14]. Recently, we confirmed that these markers are also sensitive in gastric and GEJ cancers [21]. In this study, the aim was to apply the TriMeth test to explore whether the presence of ctDNA in perioperative plasma samples is associated with a higher risk of recurrence in patients resected for gastric and GEJ AC.

## Materials and methods

### Study design

The CURE (Clinical Utility of circulating Tumor DNA in Gastro-Esophageal Cancer) study is a prospective, observational cohort study investigating the correlation between the presence of ctDNA in plasma and clinical outcomes of patients with resectable gastric and GEJ AC [22]. The Ethics Committee of the Capital Region Denmark approved the collection and use of biological samples [H-19076846]. Informed consent was obtained from all participating patients. The Danish Data Protection Agency [P-2019-701] approved the study.

### Sample size estimation

A postoperative ctDNA positivity rate of 15% and hazard ratio (HR) of 5.4 for recurrence in ctDNA-positive patients [15, 23] were assumed. Additionally, an expected median recurrence-free survival (RFS) of 30 months as reported by Al-Batran [6] was applied. Based on these assumptions, a sample size of 52 patients with complete analyses of postoperative samples was calculated using an online tool from UCSF [24]. With this sample size, a difference in RFS could be detected with an 80% power and a 5% risk of type I error [25].

### Patient population

Patients were recruited prospectively at the Department of Oncology at the Copenhagen University Hospital–Rigshospitalet, Denmark. Eligible patients were 18 years or older, diagnosed with resectable gastroesophageal carcinoma, and scheduled for perioperative chemotherapy. Four cycles of chemotherapy before surgery and four cycles of chemotherapy after surgery were planned, consisting of docetaxel, oxaliplatin, and fluorouracil/leucovorin (FLOT), in accordance with national guidelines [26, 27]. Following postoperative chemotherapy, patients were scheduled for routine clinical follow-up assessments at every 3–6 months for a duration of 2 years, as per the national guidelines [26, 27].

### Plasma samples

Blood samples were planned for collection at baseline, after one cycle of chemotherapy, after preoperative chemotherapy, and 4–6 weeks after surgery. Whole blood was collected in Streck Cell-Free DNA BCT tubes and processed within 36 h of collection. The tubes underwent centrifugation at 2250G for 10 min to separate plasma. Subsequently, the plasma was subject to another centrifugation step at 16,000G for 10 min to remove platelets and any remaining cell debris. The resulting plasma supernatant was carefully transferred to 15 mL tubes (Corning CentriStar) and stored at –80 °C until further processing. Prior to extracting cfDNA, plasma samples were thawed at room temperature and subjected to an additional centrifugation at 3000G for 10 min to separate any potential sediment from the plasma. The cfDNA extraction was performed immediately after thawing, using the QIAamp Circulating Nucleic Acid Kit (Qiagen), following the manufacturer's instructions, with a median of 8 mL (interquartile range (IQR): 7.25–8.75 mL) of plasma. The cfDNA was eluted in a total volume of 60 µL in LoBind 96-well plates (Eppendorf) and then stored at -20 °C until use.

### Sodium bisulfite conversion

Sodium bisulfite conversion was performed as previously described [19, 20]. Prior to sodium bisulfite conversion, the cfDNA eluates were dried using vacuum at 30 °C (speedVac, Concentrator plus 5350; Eppendorf AG) and resuspended in 20 µL nuclease-free water. All 20 µL of cfDNA was utilized as input for bisulfite treatment. The EZ-96 DNA Methylation-DirectTM MagPrep kit (Zymo Research) was employed for bisulfite conversion of all samples as per the manufacturer’s instructions, with adjusted volumes of reagents (60 µL CT conversion reagent, 280 µL M-Binding Buffer, 5 µL MagBinding Beads, 185 µL M-Wash Buffer, 93 µL M-Desulphonation Buffer, and 22 µL M-Elution Buffer). Positive and negative controls, including fully methylated human control DNA (Nordic Biosite) and fully unmethylated control DNA (Nordic Biosite), were incorporated in each conversion batch. Subsequently, bisulfite-converted cfDNA was analyzed using droplet digital PCR (ddPCR) within 24 h after the completion of bisulfite conversion.

### Droplet digital PCR (ddPCR)

All ddPCR experiments were performed as previously described [19, 20] and are reported in accordance with the dMIQE2020 guideline [Online Resource 1]. The analysis was conducted on a QX200 Droplet Digital PCR system (Bio-Rad) following the manufacturer's specifications. Each analysis comprised positive, negative, and no-template controls. The sample reaction mix consisted of 2–8 µL template DNA, 18 pmol forward primers, 18 pmol reverse primers, 5 pmol probes [Online Resource 2], 11 µL 2X supermix for Probes (Bio-Rad), and nuclease-free water to achieve a final reaction volume of 22 µL. On average, 20978 droplets (IQR: 20230–21456) were generated for each sample using the QX200 AutoDG Droplet Generator (Bio-Rad). Following droplet generation, samples underwent PCR amplification on a S1000 Thermal cycler (Bio-Rad) with the following program: 95 °C for 10 min, 45 cycles of 95 °C for 30 s and 56 °C for one minute, and one final cycle of 98 °C for 10 min. PCR products were stored at 4 °C for up to 12 h before being analyzed on a QX200 reader (Bio-Rad). Data analysis for ddPCR was performed using Quantasoft v1.7 software (Bio-Rad).

### DNA quantification and quality control

DNA quantification and quality control was performed as previously described [19, 20]. Prior to bisulfite conversion, assessment of cfDNA purification efficiency and lymphocyte DNA contamination was conducted using ddPCR. To determine purification efficiency, a fixed amount of soybean CPP1 DNA fragments was added to each plasma sample before cfDNA extraction. The purification efficiency was calculated as the percentage recovery of CPP1 fragments after cfDNA extraction, assessed by the CPP1 assay. Additionally, lymphocyte DNA contamination was estimated using a PBC assay, which targeted the VDJ rearranged IGH locus-specific for B cells. The median purification efficiency was 92% (IQR: 85.25–97.68). Quantification of DNA was performed before and after bisulfite conversion using a quantification assay targeting a cytosine-free region on chromosome 1 (CF assay). All assays are listed in Online Resource 2. For each sample, the DNA recovery from bisulfite conversion was calculated as the DNA quantity before bisulfite conversion divided by the DNA quantity after. The median bisulfite recovery of cfDNA samples was 51% (IQR: 44.22–56.51). Samples were excluded for further analysis if they displayed the following criteria: leucocyte DNA contamination, fewer than 10.000 droplets for ddPCR, and non-measurable CF2 signal in ddPCR after bisulfite conversion, to ensure calculation of DNA recovery.

### Methylation-specific ddPCR (TriMeth)

ctDNA assessment using TriMeth was performed at Department of Molecular Medicine, Aarhus University Hospital. The analysts were blinded to patient outcome. The TriMeth test comprises methylation-specific ddPCR assays targeting the promoter regions of the genomic regions *C9orf50*, CLIP4, and *KCNQ5* [19, 20] and the CF assay. The TriMeth ddPCR analysis was run in two duplex reactions (*C9orf50* + *KCNQ5* and CLIP4 + *CF*), as previously described [19, 20]. Each TriMeth setup included a positive control (5 ng of fully methylated DNA, Nordic Biosite), a negative control (66 ng of fully unmethylated DNA, Nordic Biosite), and a non-template control. For each ddPCR plate, the threshold for separating positive and negative droplets was objectively and automatically defined based on droplet signals in the positive and negative controls, as previously described [19, 20].

### Data analysis

A sample was considered TriMeth 'positive' if > 1 positive droplet was detected for at least 2 out of 3 TriMeth markers [19, 20]. For Trimeth (overall), and for each individual marker, we report the number of methylated copies per milliliter plasma [Online Resource 3]. To assess the predictive accuracy of the individual markers and the TriMeth sample calls, Receiver-Operating Characteristics (ROC) analyses were conducted using the R package ROCR [Online Resource 4]. RFS was the primary endpoint. RFS was calculated from the date of inclusion to the occurrence of radiological or clinical recurrence or death resulting from gastric or GEJ cancer. OS was calculated from the date of inclusion to death of any cause. Patients were censored at the end of data collection (1st of July 2023). The association of ctDNA and various prognostic variables with RFS and OS was assessed using cox proportional-hazards regression analysis. A multivariate analysis was conducted, incorporating clinical variables that exhibited statistical significance in the initial univariate analysis. Density plots and boxplots were performed using the R package ‘ggplot’. The Kaplan–Meier method and log-rank test were employed to perform survival analysis. HRs and significance were calculated using the R package 'survival', based on the time elapsed since study inclusion. All *p* values were based on two-sided testing, and differences were considered significant when *p* was less than 0.05. All statistical analyses were carried out using R software version 4.3.0.

## Results

### Patients and plasma samples

A total of 122 patients with localized, non-disseminated gastric and GEJ AC who were scheduled for perioperative chemotherapy and resection as determined by a multidisciplinary team conference, were prospectively enrolled in the study [23] between February 4th, 2020, and August 26th, 2021. All patients provided signed informed consent. Of these patients, 33 were excluded for ctDNA analysis for the following reasons: 12 due to plasma samples not being collected, 3 due to the withdrawal of informed consent, and 18 due to disease progression and/or the decision to not proceed to surgical resection.

**Fig. 1:**
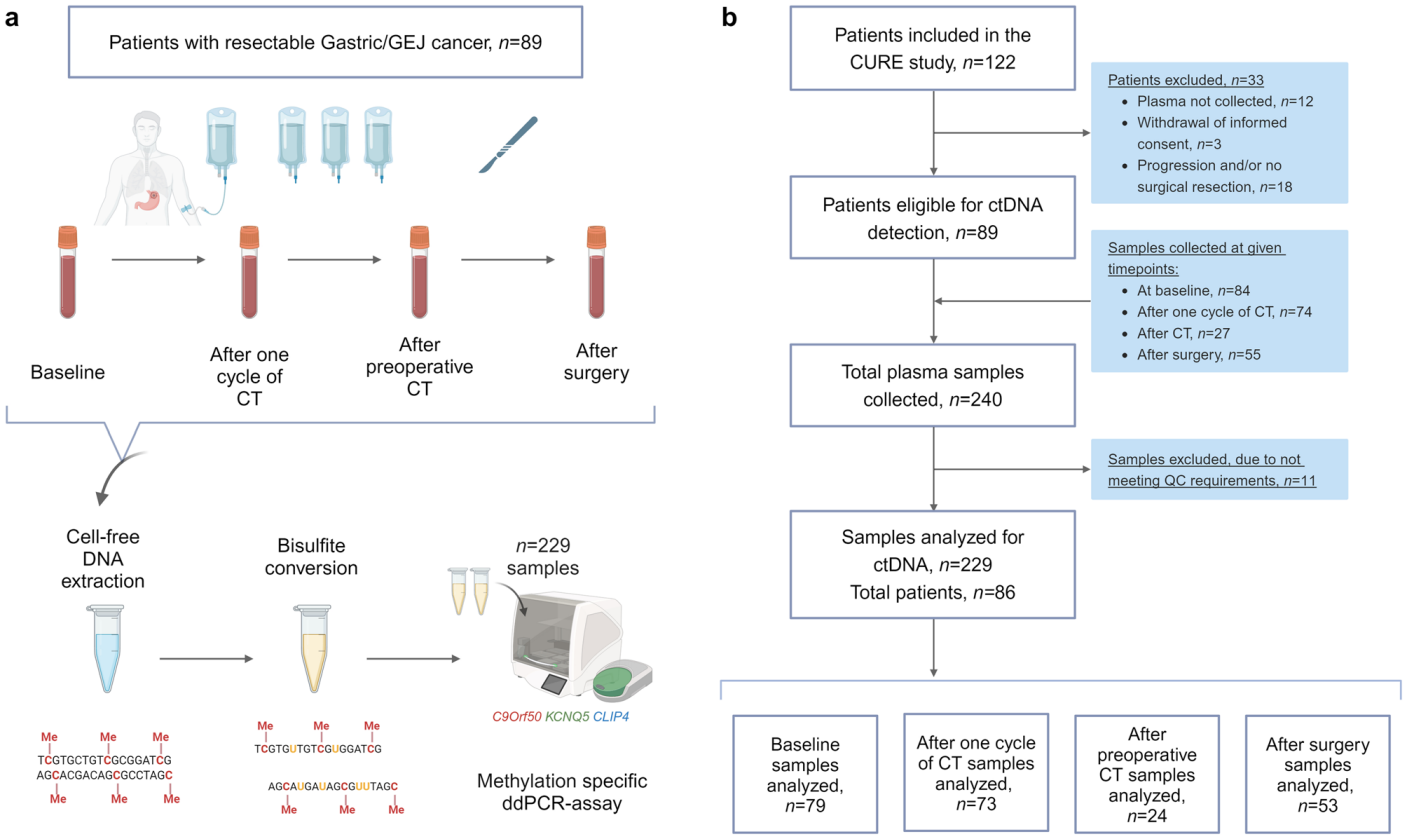
Study design. **a** Plasma samples from 86 patients with resectable gastric GEJ cancer, were collected at baseline, after one cycle of chemotherapy (CT), after preoperative CT and 2–4 weeks following surgical resection. Cell-free DNA was extracted from plasma prior to bisulfite conversion, and subsequently analyzed using a DNA methylation-specific ddPCR assay (TriMeth). Plasma samples were classified as ctDNA positive (detected) or ctDNA negative (not detected). **b** Consort diagram illustrating the patient and plasma selection process for ctDNA analysis. Figure created using BioRender.com. *GEJ* gastroesophageal junction; *CT* chemotherapy; *ddPCR* droplet digital polymerase chain reaction; *ctDNA* circulating tumor DNA, *Me* methylation

A total of 240 plasma samples were collected from the 89 patients. Plasma was collected at baseline (*n* = 84), after one cycle of chemotherapy (*n* = 74), after preoperative chemotherapy (*n* = 27), and 4–6 weeks following surgical resection (*n* = 55). Eleven plasma samples were excluded because they did not meet the sample processing quality control (QC) requirements. Consequently, the TriMeth analysis was successfully conducted on 229 plasma samples, from 86 patients. A schematic representation of the study design and a consort diagram of patient flow and sample collection are provided in Fig. 1. The TriMeth results for all plasma samples are available in Online Resource 3. The 86 patients analyzed for the presence of ctDNA had a median age of 65 years, and 84% were male. At baseline, 59% of patients had performance score (PS) of 0. In 88% of patients, the tumor localization was in the lower esophagus or GEJ. For 67% of patients, the clinical tumor stage was III–IV. The human epidermal growth factor receptor 2 (HER2) gene was amplified or overexpressed in 36%. Baseline patient characteristics are presented in Table 1.

**Table 1:** Baseline characteristics of the entire patient cohort (*n* = 86) and the subset of patients (*n* = 79) who had plasma tested for ctDNA at baseline, subdivided into ctDNA not detected (*n* = 35) and ctDNA detected (*n* = 44), and the subset of patients (*n* = 73) who had plasma tested for ctDNA after one cycle of chemotherapy, subdivided into ctDNA not detected (*n* = 46) and ctDNA detected (*n* = 27)

	All patients, *n* = 86	Patients with ctDNA detection performed at baseline, *n* = 79			Patients with ctDNA detection performed after one cycle of chemotherapy, *n* = 73		
		ctDNA not detected, *n* = 35	ctDNA detected,* n* = 44	*p* value	ctDNA not detected, *n* = 46	ctDNA detected, *n* = 27	*p* value
Age – median (IQR)	65.2 (59.0–72.0)	65.3 (59.5–71.0)	65.3 (60.6–73.4)		65.7 (59.3–72.8)	65.1 (59.5–72.0)	
Sex, *n* (%)				*p* = 0.78			***p* = 0.02**
Female	14 (16.3)	5 (14.3)	8 (18.2)		3 (6.5)	7 (25.9)	
Male	72 (83.7)	30 (85.7)	36 (81.8)		43 (93.5)	20 (74.1)	
PS at baseline, *n* (%)				*p* = 0.7			*p* = 0.76
0	51 (59.3)	19 (54.3)	28 (63.7)		29 (63.1)	15 (55.6)	
1	31 (36.0)	14 (40.0)	14 (31.8)		15 (32.6)	10 (37.0)	
2	4 (4.7)	2 (5.7)	2 (4.5)		2 (4.3)	2 (7.4)	
Location of primary tumor, *n* (%)				*p* = 0.96			*p* = 0.72
Lower esophagus	22 (25.6)	8 (22.8)	10 (22.7)		11 (23.9)	15 (55.6)	
GEJ	54 (62.8)	23 (65.8)	28 (63.7)		31 (67.4)	10 (37.0)	
Stomach	10 (11.6)	4 (11.4)	6 (13.6)		4 (8.7)	2 (7.4)	
Clinical tumor stage, *n* (%)				***p* = 0.004 **			*p* = 0.1
cT1/T2	25 (29.1)	17 (48.6)	5 (11.4)		18 (39.2)	6 (22.2)	
cT3/T4	60 (69.7)	16 (45.7)	39 (88.6)		26 (56.5)	21 (77.8)	
cTx	1 (1.2)	2 (5.7)	0 (0.0)		2 (4.3)	0 (0.0)	
Clinical nodal stage, *n* (%)				***p* = 0.03**			*p* = 0.08
cN0	56 (65.1)	27 (77.1)	24 (54.5)		34 (73.9)	14 (51.9)	
cN +	29 (33.7)	7 (20.0)	20 (45.5)		11 (23.9)	13 (48.1)	
cNx	1 (1.2)	1 (2.9)	0 (0.0)		1 (2.2)	0 (0.0)	
Metastasis stage, n (%)				–			–
cM0	81 (94.2)	32 (91.4)	44 (100.0)		42 (91.3)	26 (96.3)	
cM1	1 (1.2)	0 (0.0)	0 (0.0)		0 (0.0)	1 (3.7)	
cMx	4 (4.6)	3 (8.6)	0 (0.0)		4 (8.7)	0 (0.0)	
Lauren classification, *n* (%)				*p* = 0.7			0.86
Intestinal type	66 (76.7)	25 (71.5)	35 (79.5)		35 (76.1)	21 (77.8)	
Diffuse type	11 (12.8)	6 (17.1)	4 (9.1)		6 (13.0)	3 (11.1)	
Mixed type	8 (9.3)	4 (11.4)	4 (9.1)		4 (8.7)	3 (11.1)	
Unknown	1 (1.2)	0 (0.0)	1 (2.3)		1 (2.2)	0 (0.0)	
Signet cell carcinoma in > 50% of cells, *n* (%)				*p* = 0.9			-
Yes	2 (2.3)	1 (2.9)	1 (2.3)		0 (0.0)	0 (0.0)	
No	81 (94.2)	33 (94.2)	41 (93.2)		45 (97.8)	25 (92.6)	
Unknown	3 (3.5)	1 (2.9)	2 (4.5)		1 (2.2)	2 (7.4)	
HER2 status, *n* (%)				***p* = 0.01**			*p* = 0.3
Normal	53 (61.6)	27 (77.2)	22 (50.0)		32 (69.5)	15 (55.6)	
Positive	31 (36.1)	6 (17.1)	22 (50.0)		13 (28.3)	12 (44.4)	
No assessment	2 (2.3)	2 (5.7)	0 (0.0)		1 (2.2)	0 (0.0)	
MMR-status, *n* (%)				*p* = 0.97			*p* = 0.77
Proficient	81 (94.2)	33 (94.3)	41 (93.2)		44 (95.7)	24 (88.9)	
Deficient	1 (1.2)	0 (0.)	1 (2.3)		0 (0.0)	1 (3.7)	
No assessment	4 (4.6)	2 (5.7)	2 (4.5)		2 (4.3)	2 (7.4)	

The association between the variables was examined using a Chi-square test for independence

*ctDNA* circulating tumor DNA; *IQR* interquartile range; *PS* WHO performance score; *GEJ* gastroesophageal junction; *HER2* human epidermal growth factor receptor 2; *MMR* DNA mismatch repair

### Perioperative ctDNA detection

**Fig. 2:**
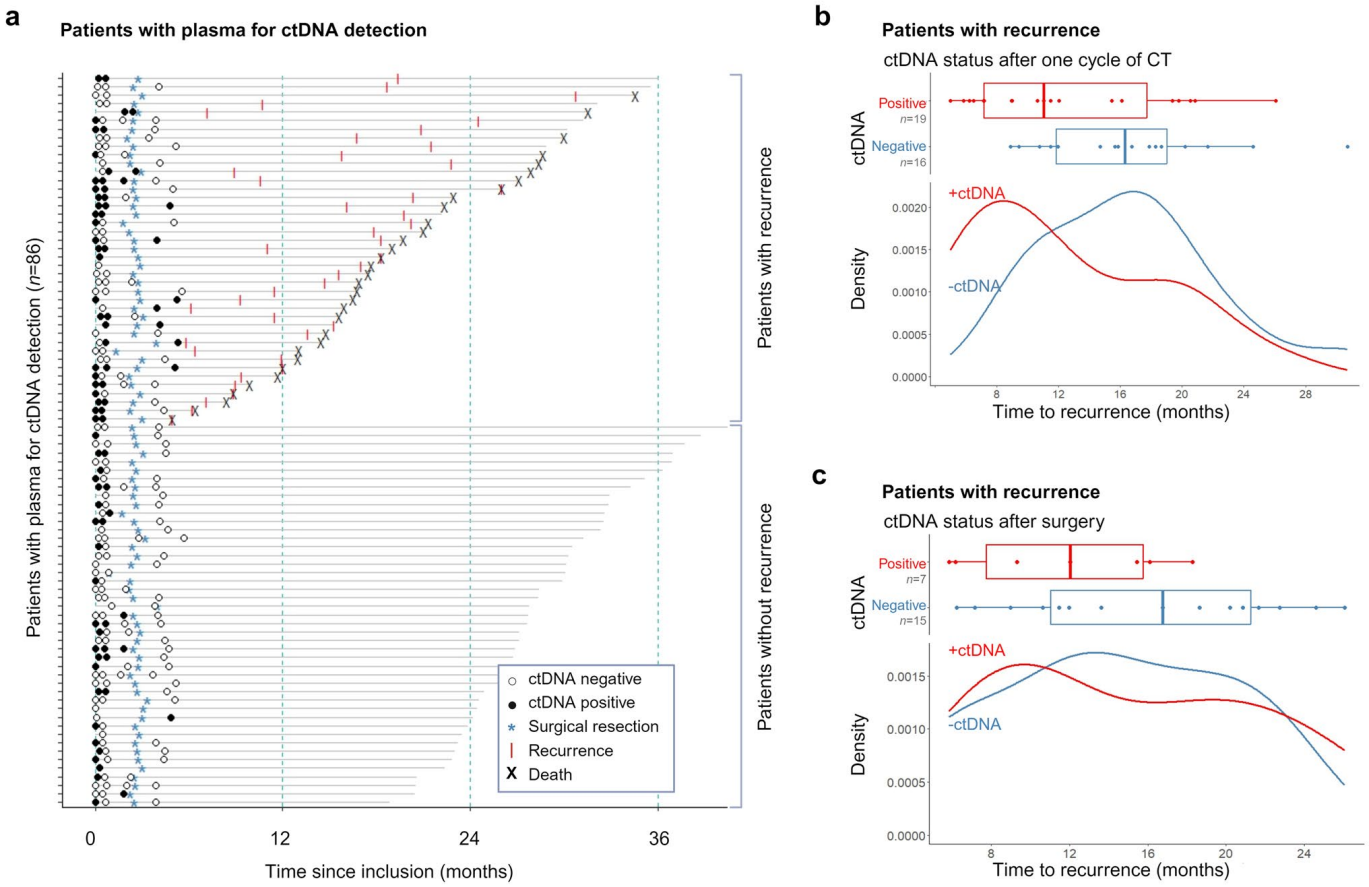
Serial plasma sampling and clinical outcomes in the 86 patients with plasma samples analyzed for ctDNA. **a** Follow-up time is indicated by the gray horizontal line. The absence or presence of circulating tumor DNA (ctDNA) is denoted by white and black dots, respectively. Surgical resection is marked with a steel blue asterisk (*), clinical or radiological recurrence with a red line (**I**), and death with a black "**X**". **b** Time to recurrence in ctDNA-positive and -negative patients after one cycle of chemotherapy (**c**) and after surgical resection, in recurrent patients (*n* = 38), presented in boxplots and a density plots. *ctDNA* circulating tumor DNA

At baseline, ctDNA was detected in 56% of the patients (44 out of 79). Patients with ctDNA detected at baseline exhibited significantly higher rates of advanced clinical tumor stages (*p* = 0.004), clinical nodal stages (*p* = 0.03), and HER2-positive tumors (*p* = 0.01) [Table 1]. After one cycle of chemotherapy, ctDNA was detected in 37% of patients (27 out of 73). ctDNA detection decreased to 25% (6 out of 24) in samples collected after completion of preoperative chemotherapy and prior to surgery. Finally, ctDNA was detected in 15% of patients (8 out of 53) after surgery. The results of all 229 serial plasma samples from the 86 patients are summarized in Fig. 2a. The time to recurrence in recurrent patients with and without ctDNA detected after one cycle of chemotherapy and after surgery, respectively, is provided in Fig. 2b, c. In the ctDNA-positive patients, we observed a shorter time to recurrence, compared to the ctDNA-negative patients.

### Overall survival and recurrence-free survival for patients with plasma eligible for ctDNA detection

**Fig. 3:**
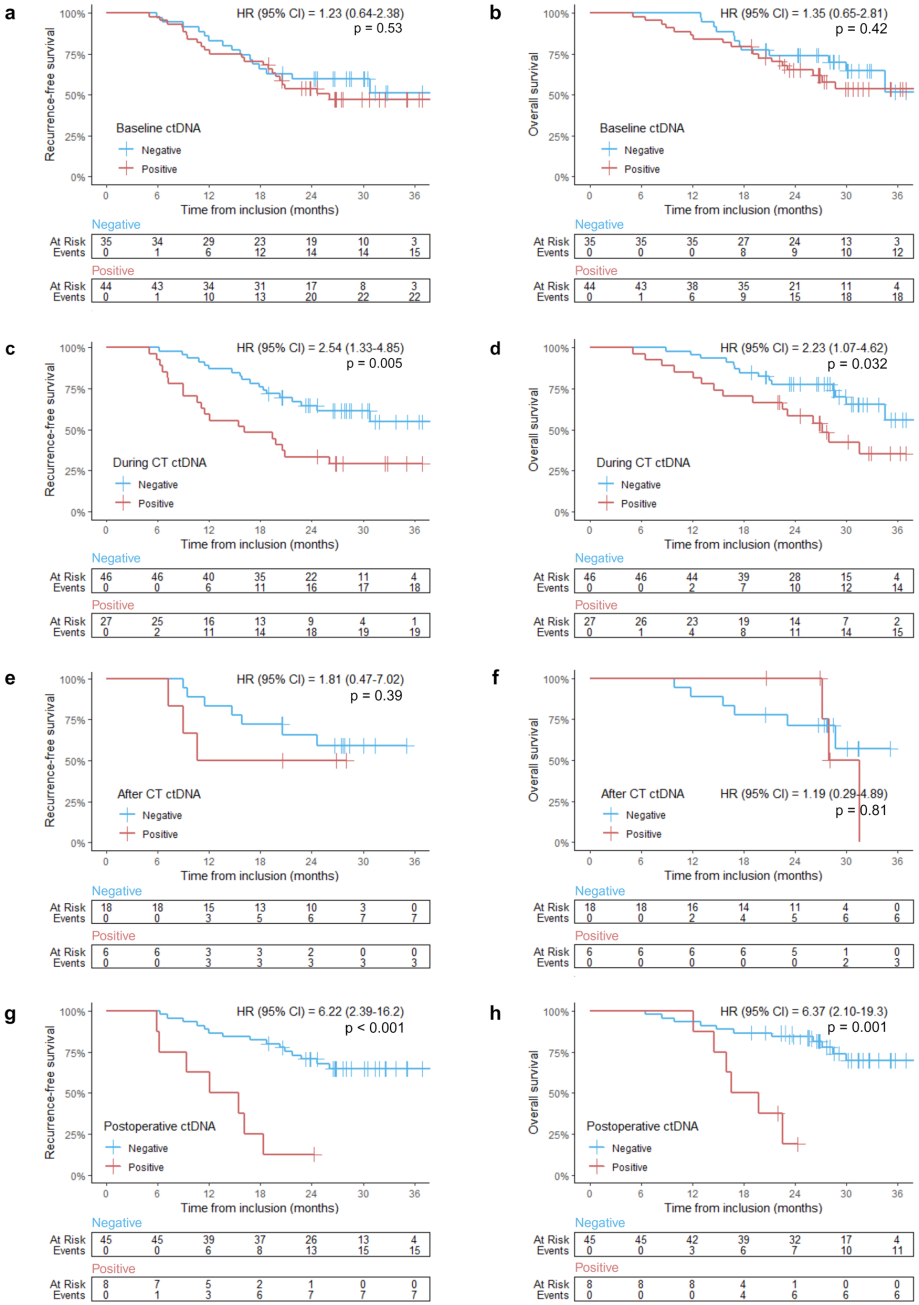
Kaplan–Meier analysis in the 86 patients with plasma analyzed for ctDNA. Red curves represent patients with detected ctDNA, while blue curves show patients without detected ctDNA. Vertical tick-marks indicate censoring. Number of events and at-risk patients are provided below each graph. **a**, **c**, **e**, **f** Recurrence-free survival (RFS) and **b**, **d**, **f**, **g** overall survival (OS) for patients with ctDNA analyses available at **a**, **b** baseline, **c**, **d** after one cycle of chemotherapy (CT), **e, f** after preoperative CT, and **g**, **h** after surgery. *HR* Hazard Ratio; *ctDNA* circulating tumor DNA; *CT* chemotherapy

The patients were followed for a median duration of 26.7 months (IQR: 23.5–28.5). By the analysis cutoff, 48% (41 out of 86) had experienced recurrence, and 37% (33 out of 86) had died, with five of these not previously having a detected recurrence. RFS and OS were compared for patients with or without ctDNA detected at four timepoints: baseline, after one cycle of chemotherapy, after preoperative chemotherapy, and after surgical resection [Fig. 3]. ctDNA detection at baseline or after preoperative chemotherapy showed no significant association with time to recurrence or death [Fig. 3a, b, e, f]. However, the presence of ctDNA after one cycle of chemotherapy was associated with a significantly reduced RFS (HR = 2.54, 95% CI 1.33–4.85, *p* = 0.005) and OS (HR = 2.23, 95% CI 1.07–4.62, p = 0.032) [Fig. 3c, d]. Patients positive for ctDNA after one cycle of chemotherapy had a 24-month RFS of 33.3% (95% CI 19.6–56.8), whereas ctDNA-negative patients had a 24-month RFS of 64.5% (95% CI 51.9–80.2). After surgery, ctDNA-positive patients had a significantly shorter RFS (HR = 6.22, 95% CI 2.39–16.2, *p* < 0.001) and OS (HR = 6.37, 95% CI 2.10–19.3, *p* = 0.001) compared to ctDNA-negative patients [Fig. 3g, h]. Patients positive for ctDNA after surgery had a 24-month RFS of 12.5% (95% CI 2.0–78.2), whereas the ctDNA-negative patients had a 24-month RFS of 70.7% (95% CI 58.4–85.5).

**Fig. 4:**
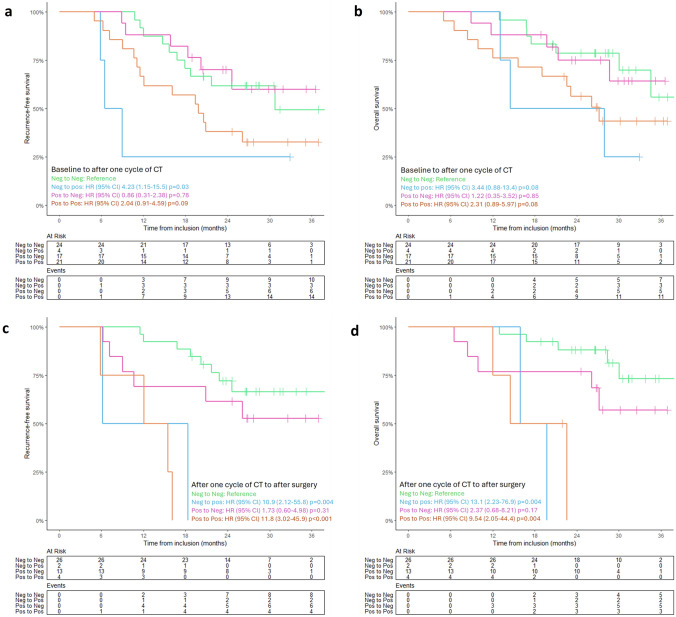
Kaplan–Meier analysis in four patient groups based on changes in ctDNA status during treatment: ‘negative to negative’ (green); ‘negative to positive’ (blue); ‘positive to negative’ (purple); ‘positive to positive’ (orange). **a**, **c** Recurrence-free survival (RFS) and overall survival (OS) (**b, d**) for ctDNA status transitions from baseline to after one cycle of chemotherapy (CT) (**a, b**) and ctDNA status transitions from after one cycle of CT to after surgical resection (**c, d**). Number of events and at-risk patients are provided below each graph. Vertical tick-marks indicate censoring. *ctDNA* circulating tumor DNA; *CT* chemotherapy

The dynamic changes in ctDNA status during treatment impacted prognosis. OS and RFS were assessed in four groups based on the transition in ctDNA status during treatment: negative to negative; positive to negative; negative to positive; positive to positive. Compared to the “negative to negative” group, patients remaining or becoming ctDNA positive postoperatively had significantly poorer OS and RFS. Prognosis was not significantly different comparing the “negative to negative” to the “positive to negative” group [Fig. 4c, d]. A similar non-significant trend was observed for the transition from baseline ctDNA status to after one cycle of chemoterapy [Fig. 4a, b].

### Regression analysis of ctDNA detection

Univariate regression analysis showed that in addition to the presence of ctDNA, factors, such as clinical nodal stage, pathological tumor stage, and pathological nodal stage, correlated with OS [Table 2]. Other clinical and pathological risk factors, including Laurén classification, signet cell carcinoma comprising more than 50% of cells, MMR-status, and resection margins, were not investigated due to the limited number of patients to meaningfully categorize into distinct groups. In the two subsequent multivariate analyses, incorporating variables with *p* < 0.05 in the univariable analysis, clinical nodal stage (HR 2.12, 95% CI 1.02–4.39, *p* = 0.05) at baseline and ctDNA after surgery (HR: 6.63, 95% CI 2.18–20.21, *p* < 0.001) and pathological tumor and nodal stages (HRs 13.8 and 3.11, 95% CIs 1.83–104.14 and 1.07–8.99 and *p* values of < 0.001 and 0.027, respectively) were found to be associated with poorer OS [Table 3].

**Table 2:** Univariate cox regression analysis for overall survival, adjusted for ctDNA status, age, gender, WHO performance score, HER-2 status, and stage at baseline, and ctDNA status, stage and tumor regression grade after surgery

Variable	OS, baseline, *n* = 79		OS, after surgery, *n* = 53	
	HR (95% CI)	*p* value	HR (95% CI)	*p* value
ctDNA				
Not detected (negative)	Reference		Reference	
Detected (positive)	1.09 (0.55–2.19)	0.80	6.63 (2.18–20.21)	** < 0.001**
Age (groups)				
< 65	Reference		–	
≥ 65	1.19 (0.57–2.49)	0.62	–	–
Gender				
Female	Reference		–	
Male	1.12 (0.43–2.93)	0.81	–	–
PS at baseline				
0	Reference		–	
1–2	1.35 (0.65–2.76)	0.41	–	–
HER-2 status at baseline				
Normal	Reference		–	
Positive	0.85 (0.42–1.93)	0.79	–	–
Clinical tumor stage				
cT1/T2	Reference		–	
cT3/T4	0.95 (0.44–2.11)	0.91	–	–
Clinical nodal stage				
cN0	Reference		–	
cN +	2.12 (1.02–4.39)	**0.05**	–	–
Pathological tumor stage				
ypT1/T2	–		Reference	
ypT3/T4	–	–	13.8 (1.83–104.14)	** < 0.001**
Pathological nodal stage				
ypN0	–		Reference	
ypN +	–	–	3.11 (1.07–8.99)	**0.027**
TRG				
TRG1-2	–		Reference	
TRG3-5	–	–	2.01 (0.65–6.20)	0.20

*p* values in bold indicate statistical significance (*p* ≤ 0.05)

*OS* overall survival; *HR* hazard ratio; *ctDNA* circulating tumor DNA; *PS WHO* performance score; *HER-2* human epidermal growth factor receptor 2; *TRG* tumor regression grade according to Mandard

**Table 3:** Multivariate cox regression analysis for overall survival, adjusted for prognostic factors at baseline, after one cycle of chemotherapy and after surgery, respectively, with p values < 0.05 in the univariate regression analysis (clinical nodal stage, ctDNA status, pathological tumor stage, and pathological nodal stage)

Variable	OS, baseline, *n* = 79		OS, after one cycle of chemotherapy, *n* = 73		OS, after surgery, *n* = 53	
	HR (95% CI)	*p* value	HR (95% CI)	*p* value	HR (95% CI)	*p* value
ctDNA status						
Not detected (negative)	Reference		Reference		Reference	
Detected (positive)	1.02 (0.45–1.97)	0.90	2.40 (1.09–5.26)	**0.03**	7.33 (2.39–22.47)	** < 0.001**
Clinical nodal stage						
cN0	Reference		Reference		–	
cN +	2.18 (1.05–4.55)	**0.04**	1.70 (0.77–3.72)	0.18	–	**–**
Pathological tumor stage						
ypT1/T2	–		–		Reference	
ypT3/T4	–	–	–	–	12.63 (1.63–98.93)	**0.02**
Pathological nodal stage						
ypN0	–				Reference	
ypN +	–	–	–	–	1.51 (0.51–4.50)	0.45

*p* values in bold indicate statistical significance (*p* ≤ 0.05)

*OS* overall survival; *HR* hazard ratio; *ctDNA* circulating tumor DNA

## Discussion

This prospective study demonstrated that ctDNA detection in plasma, using a tumor-agnostic approach is a robust and independent prognostic marker in resectable gastric and GEJ AC. Interestingly, the presence of ctDNA after just one cycle of preoperative chemotherapy was associated with significantly shorter OS and RFS. This finding suggests that valuable information about patient prognosis can be obtained at an early stage during preoperative cancer treatment. Early clearance of ctDNA by chemotherapy could have the potential to serve as a preoperative tool to predict the benefit of surgery. After surgical resection, the presence of ctDNA in plasma was also significantly correlated with a shorter OS and RFS. Additionally, we observed that patients who became or remained ctDNA positive over the course of treatment had a shorter OS and RFS, while patients who were ctDNA negative from baseline and remained ctDNA negative or became ctDNA negative had a longer OS and RFS. These findings underscore the prognostic value of dynamic changes in ctDNA status during treatment, demonstrating that persistent ctDNA positivity is associated with significantly poorer OS and RFS. Notably, patients transitioning from a positive to negative ctDNA status did not exhibit a significantly different prognosis compared to those who were consistently ctDNA negative, suggesting that clearance of ctDNA by chemotherapy may mitigate adverse prognostic implications associated with initial positivity.

Our results highlight the potential role of ctDNA as a dynamic biomarker for monitoring treatment response and as a tool for predicting recurrence after the completion of curative treatment. Cox multivariate analysis confirmed the independent prognostic value of ctDNA detection after one cycle of chemotherapy and post-surgery.

A tumor-agnostic strategy was applied to detect ctDNA using a multiplex ddPCR assay targeting the DNA methylation markers *C9orf50*, *KCNQ5*, and CLIP4. The assay was initially developed for detection of CRC and the three methylation markers were selected based on DNA methylation array data from > 5000 samples, including tumor samples from 17 different cancer types, normal colorectal mucosa, and blood samples [19, 20]. In addition to being highly methylated in CRCs, the markers are also methylated in gastric and GEJ ACs, as we and others have recently reported [21, 28]. The sensitivities of the markers in baseline samples of gastric and GEJ cancer patients (56%), reported here, are lower compared to the previous reported sensitivities in CRC baseline samples (63–86% for non-metastatic patients) [19, 20]. This could potentially impact the clinical utility of the markers in gastric and GEJ cancer patients. In our previous work [21], we showed that *KCNQ5* and *C9orf50* methylation was present in all the assessed gastric and GEJ tumors, whereas CLIP4 methylation was present in only 75% of gastric and GEJ tumors. This discrepancy highlights the potential need to replace CLIP4 or adjust the TriMeth scoring algorithm to improve the sensitivity toward gastric and GEJ cancers. Although a CLIP4 signal in plasma suggests the presence of tumor DNA when corroborated by *KCNQ5* or *C9orf50*, the suboptimal performance of CLIP4 in gastric and GEJ cancers should be noted. However, when comparing the prognostic power of ctDNA after end of treatment (chemotherapy and surgery), the HR reported here (HR: 6.22, 95% CI: 2.4–16.2) are comparable to what we report in CRC (HR: 7.3, 95% CI: 3.8–14.7) [14], highlighting the potential of the test in gastric and GEJ cancers.

A few prospective and retrospective studies have highlighted the potential of ctDNA detection as a tool for detecting cancer recurrence after surgery in patients with gastric cancers [16, 29–31]. Notably, these studies utilized tumor-informed next-generation sequencing (NGS) methods. In a recent retrospective study [32], ctDNA's role as a recurrence risk biomarker was explored in 212 patients with esophageal and gastric cancers who had undergone curative therapy. Another recent study that prospectively investigated ctDNA as a biomarker in 63 patients with locally advanced resectable gastric and GEJ cancer [33] found that persistent ctDNA positivity is linked to poor outcomes. Both studies [32, 33] reported a strong association between ctDNA positivity and shorter RFS. Although these studies used tumor-informed methods for ctDNA detection, their findings, complement our results, affirming the relevance of a tumor-agnostic method. Tumor-agnostic ctDNA analysis offers several advantages, including standardized testing, faster turnaround times, simplified logistics, and cost reduction by eliminating the need for prior tumor analysis. These positive aspects underscore the practicality and efficiency of a tumor-agnostic approach.

While we report very promising findings, a few limitations of our study should be acknowledged. First, the sample size is relatively small, underscoring the need for larger cohorts and external validation to confirm the robustness and generalizability of our findings. However, it is important to note that our sample size was determined based on a pre-study power calculation, ensuring that the sample size is sufficient to answer the primary objective of this study. Additionally, the collection of only a limited number of plasma samples after preoperative chemotherapy is a potential concern, as it could have affected the accuracy of the ctDNA analysis at this timepoint.

## Conclusions

Our study demonstrates that tumor-agnostic ctDNA detection methods can offer valuable prognostic insights for patients with gastroesophageal ACs, treated with curative intent. The ability to assess risk of recurrence and death, holds promise for personalized treatment strategies and improve disease management. Clinical randomized trials are needed for confirmation, and future studies should explore ctDNA-guided approaches to improve patient management.

## Supplementary Information

Below is the link to the electronic supplementary material.

Supplementary file1 (XLSX 29 KB)

Supplementary file2 (PDF 88 KB)

Supplementary file3 (PDF 98 KB)

Supplementary file4 (XLSX 29 KB)

## Data Availability

The original data presented in the study are included in the Supplemental Material. Further inquiries can be directed to the corresponding author.
