# Supplementary material for: Circulating tumor DNA predicts recurrence and survival in patients with resectable gastric and gastroesophageal junction cancer
Source: Gastric Cancer. 2024 Oct 5;28(1):83–95. doi: 10.1007/s10120-024-01556-9 (PMC11706848; doi:10.1007/s10120-024-01556-9)
Supplement: Supplementary file 2 — Supplementary file2 (PDF 88 KB) [file 10120_2024_1556_MOESM2_ESM.pdf]

Supplementary Figure 1. Receiver Operating Characteristic (ROC) curve displaying performance of each methylated marker, TriMeth, and 10 Healthy Controls. True Positive (TP) cases represent all baseline samples, while True Negative (TN) cases include post-operative samples without death or recurrence events.

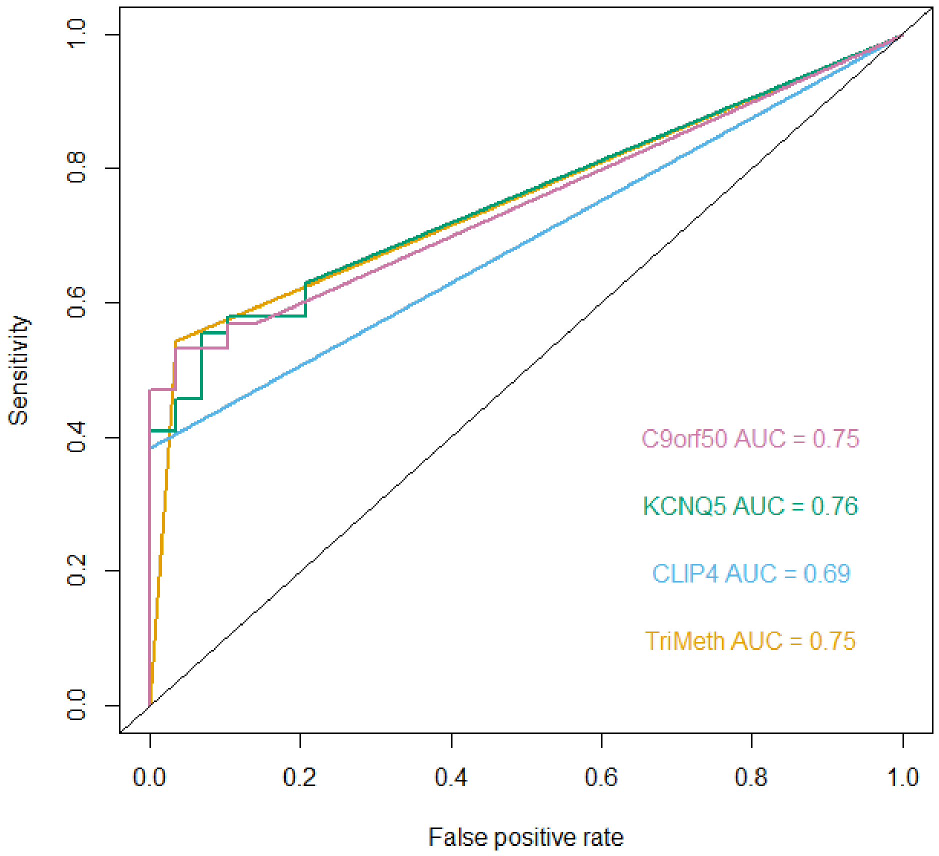

Article title: Circulating tumor DNA predicts recurrence and survival in patients with resectable gastric and gastroesophageal junction cancer

Journal name: Gastric Cancer

Author names and affiliations:

Cecilie Riis Iden (MD)1, Salah Mohammad Mustafa (graduate student)2,3, Nadia Øgaard (MSc, PhD)2,3, Tenna Henriksen (MSc, PhD) 2,3 Sarah Østrup Jensen (MSc, PhD)2,3, Lise Barlebo Ahlborn (MSc, PhD)4, Kristian Egebjerg (MD)1, Lene Baeksgaard (MD, PhD)1, Rajendra Singh Garbyal (MD)5, Mette Kjoelhede Nedergaard (MD, PhD) 5, Michael Patrick Achiam (MD, PhD)6, Claus Lindbjerg Andersen (MSc, PhD)2,3, Morten Mau-Sørensen (MD, PhD)1

1 Department of Oncology, Copenhagen University Hospital, Rigshospitalet, Blegdamsvej 9, 2100 Copenhagen, Denmark

2 Department of Molecular Medicine, Aarhus University Hospital, Palle Juul-Jensens Boulevard 99, 8200 Aarhus N, Denmark

3 Institute of Clinical Medicine, Faculty of Health, Aarhus University, Palle Juul-Jensens Boulevard 82, 8200 Aarhus N, Denmark

4 Department of Genomic Medicine, Copenhagen University Hospital, Rigshospitalet, Blegdamsvej 9, 2100 Copenhagen, Denmark

5 Department of Pathology, Copenhagen University Hospital, Rigshospitalet, Blegdamsvej 9, 2100 Copenhagen, Denmark

6 Department of Surgery & Transplantation, Copenhagen University Hospital, Rigshospitalet, Blegdamsvej 9, 2100 Copenhagen, Denmark

E-mail address of the corresponding author: paul.morten.mau-soerensen@regionh.dk
